# Tenofovir vs. entecavir in chronic hepatitis B: A retrospective cohort study of hepatocellular carcinoma risk in a tertiary hospital in southern Brazil

**DOI:** 10.1016/j.bjid.2025.104603

**Published:** 2025-12-17

**Authors:** Adriana Neis Stamm, Cristiane Valle Tovo, Andressa Noal, Camila Ubirajara Silva, Jaysa Pizzi, Pedro Moreno Fonseca, Dimas Alexandre Kliemann

**Affiliations:** a Hospital Nossa Senhora da Conceição, Porto Alegre, RS, Brazil; b Universidade Federal de Ciências da Saúde de Porto Alegre (UFCSPA), Porto Alegre, RS, Brazil; c Universidade Federal de Ciências da Saúde de Porto Alegre (UFCSPA), Internal Medicine Department and Graduate Program in Hepatology, Porto Alegre, RS, Brazil; d Hospital Sanatório Partenon, Injectable Medication Application and Monitoring Center, Porto Alegre, RS, Brazil; e Hospital Santa Casa de Porto Alegre, Porto Alegre, RS, Brazil

**Keywords:** Hepatitis b, Hepatocellular carcinoma, Tenofovir, Entecavir, Antiviral therapy

## Abstract

**Background:**

Chronic Hepatitis B Virus (HBV) infection remains a major global health burden, affecting approximately 296 million individuals worldwide and leading to significant morbidity and mortality due to cirrhosis and Hepatocellular Carcinoma (HCC). Although Nucleos(t)ide Analogues (NAs) such as Tenofovir Disoproxil Fumarate (TDF) and Entecavir (ETV) effectively suppress HBV replication, their comparative efficacy in reducing HCC risk remains controversial.

**Methods:**

This retrospective cohort study analyzed HBV-monoinfected patients treated with either TDF or ETV at a tertiary hospital in southern Brazil between 2014 and 2021. Patients with co-infections (HIV, HCV), prior HCC diagnosis, liver transplantation, or others antiviral treatments, like lamivudine e/ou alfainterferon, were excluded. Data on demographics, treatment regimens, disease progression, and HCC incidence were extracted from institutional databases. Statistical analyses included Fisher's exact test and Poisson regression to determine Relative Risk (RR) and 95 % Confidence Intervals (95 % CIs).

**Results:**

Of the 127 included patients, 66 (52 %) received TDF and 61 (48 %) received ETV. Over a 7-year follow-up period, 10 patients developed HCC – 8 in the ETV group (13.1 %) and 2 in the TDF group (3 %). In the raw analysis, TDF use was associated with a significantly lower risk of HCC progression (RR = 0.23, *p* = 0.057, 95 % CI: 0.05‒1.046). After adjusting for the variable’s cirrhosis/advanced fibrosis and age in the multivariate analysis, this association lost statistical significance (RR = 0.33, *p* = 0.18, 95 % CI: 0.068‒1.685). This indicates that the apparent protective effect of tenofovir may have been influenced by these variables and may have limited the statistical power of the adjusted model. Patients receiving ETV had a higher prevalence of advanced liver disease, including cirrhosis (64.7 % vs. 35.3 %, *p* < 0.05), hepatic encephalopathy (7 % vs. 2.4 %, *p* < 0.05), and portal hypertension (12.5 % vs. 3.4 %, *p* < 0.05). The incidence rate of HCC was 1.12 per 100 person-years (to be interpreted with caution due to limited follow-up data).

**Conclusion:**

Treatment with TDF was associated with a lower risk of HCC compared to ETV in the bivariate analysis, but this association lost significance in the multivariate analysis. These findings suggest that the initially observed protective effect of TDF against hepatic carcinogenesis may have been partially explained by confounding factors (cirrhosis/advanced fibrosis and age), as well as reflecting the limited sample size. Further studies are warranted to provide a more robust comparative evaluation of antiviral therapies.

## Introduction

Approximately 296 million people worldwide live with hepatitis B, including about 1 million in Brazil. The Hepatitis B Virus (HBV) remains a leading cause of liver cirrhosis and Hepatocellular Carcinoma (HCC).^1^^,^^2^ The persistent replication of HBV is the primary factor driving the progression from Chronic Hepatitis B (CHB) to cirrhosis and HCC.^2^ Long-term therapy with Nucleos(t)ide Analogues (NAs) can suppress HBV replication, reducing the risk of HCC and mortality in patients with CHB.^3^ According to current international practice guidelines, Tenofovir Disoproxil Fumarate (TDF), Tenofovir Alafenamide (TAF), and Entecavir (ETV) are recommended as first-line antiviral agents for CHB due to their high antiviral efficacy and low resistance rates.^3^, ^4^, ^5^

Although few small randomized trials have compared the incidence of HCC between ETV-treated and TDF-treated HBV patients, recent data from observational cohort studies and meta-analyses suggest that the comparative HCC risk remains controversial.^6^^,^^7^ Some researchers believed that patients receiving TDF had a lower risk of HCC,^8^, ^9^, ^10^ while others argued that there was no difference in the efficacy of the two drugs.^11^^,^^12^ However, it remains unclear whether there are differences in the effectiveness of these drugs in preventing disease progression.

The primary aim of this study was to compare antiviral therapies with Tenofovir and Entecavir in the development of HCC between the years 2014 and 2021 in people with chronic hepatitis B in south of Brazil. Secondary aims included identifying predictors of HCC development, such as cirrhosis and advanced liver fibrosis.

## Materials and methods

This retrospective cohort study included patients diagnosed with chronic Hepatitis B Virus (HBV) infection between January 2014 and December 2021 at *Grupo Hospitalar Conceição* (GHC), a tertiary public hospital in southern Brazil. All patients notified with viral hepatitis during this period were evaluated. Data regarding age, sex, antiviral therapy, HIV and Hepatitis C Virus (HCV) co-infection, cirrhosis, and Hepatocellular Carcinoma (HCC) were collected from the *Notifiable Diseases Information System* (Sistema de Informação de Agravos de Notificação ‒ SINAN) for Viral Hepatitis, the *Logistics Information and Control System (Sistema de Informação e Controle* Log*ístico* ‒ SICLON) and the GHC electronic medical records.

A list comprising >60 infectious diseases, including viral hepatitis, is subject to mandatory reporting in Brazil. The notification of mandatory-reportable diseases is a legal obligation and does not violate ethical principles. Failure to report constitutes a public health infraction and may result in penalties. Data on reported diseases are stored in the SINAN. Regarding viral hepatitis, initial reporting does not differentiate between virus types; definitive classification requires the completion of an epidemiological investigation to confirm (or exclude) the specific viral etiology. In the present study, only records with finalized investigations were accessed.

The inclusion criteria encompassed patients aged 18-years or older with a confirmed diagnosis of chronic HBV infection, defined as the presence of positive HBsAg or a detectable HBV viral load with positive anti-HBc for more than six months. Patients who had received treatment with Tenofovir (TDF), Entecavir (ETV) for at least one year were included in the study.

All patients with HIV infection (confirmed by a reactive anti-HIV test or detectable HIV viral load), HCV monoinfection, HBV/HCV coinfection, those without confirmatory data for HBV, with previous diagnosis of HCC or hepatic transplantation and those receiving treatment with lamivudine and/or pegylated interferon or not undergoing antiviral therapy were excluded from the analysis.

Although all included patients had a confirmed diagnosis of chronic Hepatitis B Virus (HBV) infection, medical records did not always provide the exact year when this diagnosis had been established. Similarly, variables such as HBeAg status and HBV viral load were not systematically available in all cases. The diagnosis of Hepatocellular Carcinoma (HCC) was obtained from clinical records or confirmation based on imaging methods ‒ computed tomography or magnetic resonance imaging ‒ without a systematic description of the use of the LI-RADS classification and the exact year of detection was not consistently documented. Likewise, information regarding the duration of antiviral therapy prior to HCC diagnosis was often missing, which limited further analysis of this aspect. For the definition of cirrhosis or advanced fibrosis, findings reported in medical records and imaging studies, including ultrasound, computed tomography, and magnetic resonance imaging, were considered.

The statistical analysis was conducted using IBM SPSS software, version 25. Descriptive analysis for treatment outcomes, hepatopathy, and Hepatocellular Carcinoma (HCC) was performed using measures of absolute and relative frequencies, central tendency, and dispersion. Categorical variables were presented as absolute numbers (n) and relative percentages ( %). For bivariate analysis, Fisher's exact test was employed to assess associations, with Relative Risk (RR) and corresponding 95 % Confidence Intervals (95 % CIs) calculated. A significance level of 5 % (α = 0.05) was adopted, with *p* < 0.05 considered statistically significant. Comparisons of proportions between independent groups were conducted using the Chi-Square test or Fisher's exact test for nonparametric data.

To control for potential confounding factors, a multivariate analysis was performed using Poisson regression with robust variance, including independent variables identified in the bivariate analysis. Adjusted Relative Risk (adjusted RR) was used as the measure of association.

The study received ethical approval from the *Grupo Hospitalar Conceição* (GHC) Research Ethics Committee under protocol number 61,185,722.6.0000.5530. The requirement for informed consent was statement, as the observational study utilized only data from medical records and institutional information systems. All data were handled confidentially, and the results were reported in a manner that prevents individual identification of participants.

## Results

**Fig. 1:**
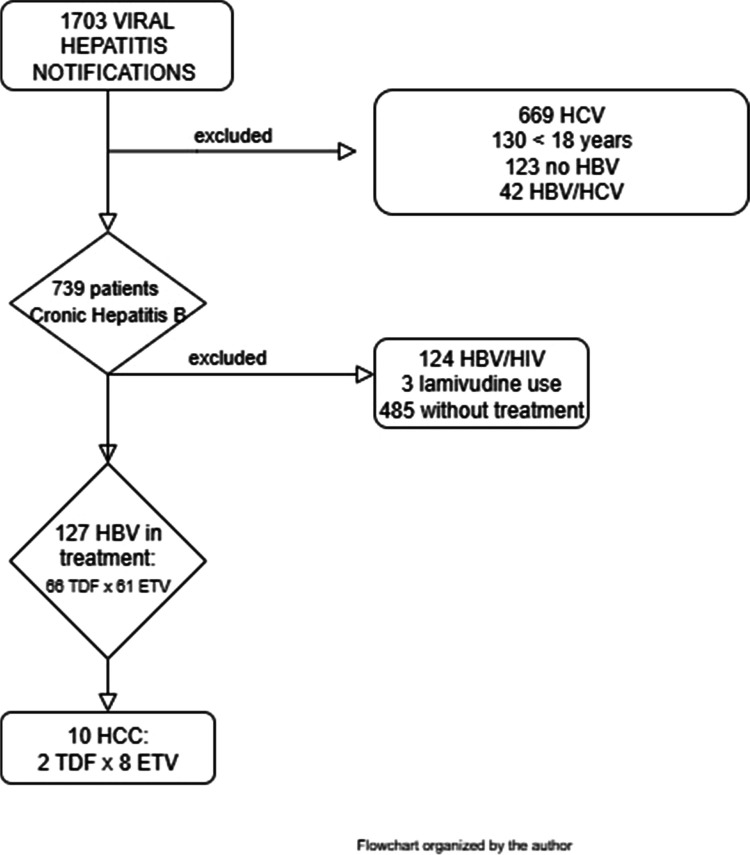
Flowchart. Patients with SINAM notification for viral hepatitis between 2014‒2021.

Data were collected from 1703 individuals with viral hepatitis reported in the SINAN system. The following individuals were excluded from the analysis: 485 who were not on antiviral therapy, 124 also diagnosed with HIV, 130 patients under 18-years of age, 669 with an exclusive diagnosis of HCV, 42 with HCV/HBV coinfection, 123 without confirmatory data and 3 receiving other treatments than TDF or ETV. Consequently, 127 individuals with hepatitis B were included in the analysis (Fig. 1).

Table 1 presents the sociodemographic profile of the patients, including sex and age. Among the participants, 39 (30.7 %) were aged over 60-years and 75 (59.1 %) were male. Patients receiving TDF were generally younger (< 60-years), predominantly male, and had a lower risk of HCC.

**Table 1:** Demographic and clinical characteristics of the sample.

Variable	n	%	Mean ± SD	Median (min–max)	Mode
Age (years)			51.8 ± 13.6	47.5 (19–85)	53
18–29	8	6.3			
30–39	14	11			
40–49	33	26			
50–59	33	26			
60–69	26	20.5			
≥ 70	13	10.2			
Sex					
Female	52	40.9			
Male	75	59.1			
Treatment					
Tenofovir	66	52			
Entecavir	61	48			
Overall mortality					
Yes	12	9.4			
No	115	90.6			

Among the 127 HBV-monoinfected patients, 52 % (66) received TDF and 48 % (61) received ETV. During a 7-year follow-up period, 10 patients developed HCC: 2 in the TDF group and 8 in the ETV group. The incidence of HCC was significantly higher in first analysis in the ETV group (13.1 %) than in the TDF group (3 %) (*p* = 0.057; 95 % CI: 0.051–1.046), with all cases occurring in male patients (*p* < 0.05) within the same age group.

A higher proportion of patients with advanced liver disease was observed in the ETV group, as indicated by the prevalence of cirrhosis/advanced fibrosis (64.7 % in the ETV group vs. 35.3 % in the TDF group; *p* < 0.05) (Table 2). Similar trends were observed for hepatic encephalopathy (19.5 % in the ETV group vs. 1.2 % in the TDF group) and portal hypertension (34.1 % in the ETV group vs. 2.3 % in the TDF group), suggesting more advanced liver disease in the ETV group (Table 2).

**Table 2:** Advanced Liver disease.

	Tenofovir	Entecavir	p
Cirrhosis / Advanced Fibrosis	12 (35.5 %)	22 (64.7 %)	0.028
Ascites	4 (2.3 %)	16 (34.1 %)	<0.05
Encephalopathy	3 (1.2 %)	9 (19.5 %)	<0.05
Variceal Bleeding	5 (2.9 %)	15 (31.7 %)	<0.05
Portal Hypertension	4 (2.3 %)	16 (34.1 %)	<0.05
Hepatocellular carcinoma	2 (3 %)	8 (13.1 %)	0.057

Among the 127 patients evaluated, 10 new cases of HCC were identified, corresponding to a cumulative incidence of 7.9 % over the 7-year period (2014‒2021). Given the lack of confirmed longitudinal follow-up for patients without HCC, the estimated incidence rate per person-time (1.12 per 100 person-years) should be interpreted with caution, since the actual time at risk could not be accurately determined. Regarding the secondary outcome, the incidence of cirrhosis was higher in the Entecavir (ETV) group (64.7 %) compared to the Tenofovir (TDF) group (35.3 %), as shown in Table 3.

**Table 3:** Secondary outcome.

	Tenofovir	Entecavir	p
Group Age			
18‒29	06 (9.1 %)	02 (3.3 %)	‒
30‒39	07(10.6 %)	07 (11.5 %)	‒
40‒49	19 (28.8 %)	14 (22.9 %)	‒
50‒59	17 (25.7 %)	16 (26.2 %)	‒
60‒69	10 (15.1 %)	16 (26.2 %)	‒
> 70	07 (10.6 %)	06 (9.8 %)	
Sex			
Male	34 (51.5 %)	41 (67.2 %)	0.053
Treatment Used	66 (52 %)	61 (48 %)	‒
Cirrhosis / Advanced Fibrosis	12 (35.3 %)	22 (64.7 %)	0.028
Hepatocellular carcinoma	02 (3 %)	09 (13.1 %)	0.057

For the primary endpoint, a reduction in the risk of progression to Hepatocellular Carcinoma (HCC) was observed in the bivariate analysis in the TDF group compared to the ETV group (RR = 0.23, 95 % CI 0.05–1.046, *p* = 0.057). Over the seven-year follow-up period, patients receiving TDF demonstrated a 77 % lower gross risk of HCC progression. However, when adjusted for potential confounders such as age and cirrhosis in the multivariate analysis, this apparent protective effect of TDF was no longer statistically significant (adjusted RR = 0.33, 95 % CI 0.068–1.685 *p* = 0.186). These results suggest that, despite the trend toward reduced HCC incidence in the TDF group, our study could not confirm the superiority of TDF over ETV in preventing hepatocarcinogenesis, as shown in Table 4.

**Table 4:** Primary and secondary outcomes: bivariate and multivariate analysis.

Variable	Bivariate – RR (95 % CI)	p-value	Multivariate – Adjusted RR (95 % CI)	p-value
Age	0.826 (0.340–3.864)	0.05	0.97 (0.940–1.014)	0.213
Cirrhosis / Advanced fibrosis	10.94 (2.44–48.980)	<0.05	8.99 (1.792–44.905)	<0.05
Treatment: TDF vs. ETV	0.23 (0.05–1.046)	0.057	0.33 (0.068–1.685)	0.186

## Discussion

The introduction of main antiviral agents, the nucleos(t)ide analogues, especially TDF and ETV, has markedly reduced the incidence of hepatocellular carcinoma among patients with chronic hepatitis B. Both agents are considered effective first-line therapies due to their strong antiviral potency and high genetic barrier to resistance.^3^^,^^4^ The present study, conducted in a tertiary hospital in southern Brazil, evaluated outcomes in a non-Asian population accounted for key variables such as cirrhosis and duration of follow-up.

Data from large Asian cohorts have frequently indicated a lower risk of HCC among patients treated with TDF compared to ETV, although the exact mechanisms underlying this apparent superiority is unclear, such studies as the nationwide Korean cohort by Choi J et al. reporting a significant reduction in risk.^9^ Subsequent evidence involving large populations also suggested results favoring TDF, perhaps due to faster and more pronounced virological suppression compared to ETV.^13^, ^14^, ^15^, ^16^ Nonetheless, this apparent benefit has not been consistently demonstrated. More detailed analyses have suggested that the advantage of TDF may be limited to specific subgroups, such as HBeAg-positive individuals and patients with compensated cirrhosis.^6^^,^^7^^,^^17^^,^^18^ Furthermore, studies have shown comparable incidences of HCC between both agents, independent of clinical variables.^11^^,^^12^

On the other hand, evidence from Caucasian cohorts present therapeutic equivalence in all subgroups. The multicenter European PAGE-B study and the Hepather cohort both demonstrated no significant differences in HCC incidence between TDF and ETV, reinforcing that in Western populations the two agents provide similar protection, even in male patients and with cirrhosis.^19^^,^^20^ Reflecting these observations, international guidelines do not recommend one drug over the other when the prevention of HCC is the primary consideration.^3^^,^^5^

Our results showed, although an apparent protective effect of TDF over ETV was observed in initial analyses, this advantage was lost after adjustment for confounders such as cirrhosis and advanced fibrosis. This suggests that the differences may be more reflective of patient baseline characteristics than intrinsic drug efficacy.

We acknowledge certain limitations, first incomplete data in medical records and loss of patients over the time may have introduced bias. Second, the follow-up period, although clinically relevant, was relatively short to access the long-term risk of HCC. Third, treatment allocation was influenced by the recommendations of the Brazilian guideline for Hepatitis B, cirrhotic patients were indicated to use entecavir while patients without cirrhosis and also younger patients used tenofovir, a fact that may have impacted the primary outcome analyzed.

In conclusion, this study supports the evidence suggesting therapeutic equivalence between TDF and ETV in reducing HCC risk, particularly after adjustment for baseline disease characteristics. While TDF may appear superior in certain subgroups,^8^, ^9^, ^10^^,^^13^, ^14^, ^15^, ^16^ this effect is not consistent across all populations.^6^^,^^7^^,^^17^^,^^18^ Future research should focus on randomized controlled trials directly comparing antiviral therapies, including Tenofovir Alafenamide (TAF), and widely, prospective cohorts with extended follow-up. Such studies are essential to refine estimates of HCC incidence, to improve the identification of prognostic factors, and to clarify the long-term impact of antiviral treatment on the natural history of chronic hepatitis B, preventing hepatocarcinogenesis.

## Funding

This research did not receive any specific grant from funding agencies in the public, commercial or not-for-profit sectors.

## Data availability

The datasets generated during this study are available from the corresponding author upon reasonable request.

## Conflicts of interest

The authors declare no conflicts of interest.
